# Impact of Hospital Volume on Outcome After Surgical Treatment for Hydrocephalus: A U.S. Population Study From the National Inpatient Sample

**DOI:** 10.7759/cureus.13617

**Published:** 2021-02-28

**Authors:** Majid Khan, Brian Farnsworth, Brandon R Pope, Brandon Sherrod, Michael Karsy

**Affiliations:** 1 Department of Medicine, Reno School of Medicine, University of Nevada, Reno, USA; 2 Department of Neurosurgery, University of Utah, Salt Lake City, USA

**Keywords:** hydrocephalus, shunt, incidence, prevalence, epidemiology, disposition, cost

## Abstract

Introduction

Hydrocephalus remains a common condition with significant patient morbidity; however, accurate accounting of the incidence of this disease as well as of the impact of hospital volume on outcome remains limited.

Methods

The National Inpatient Sample was used to evaluate patients who underwent surgical treatment of hydrocephalus from 2009-2013. Patient demographics (e.g., length of stay, disposition, charges), and the impact of hospital volume on outcomes were evaluated.

Results

A total of 156,205 patients were identified. Ventriculoperitoneal (VP) shunting the most common type of device (35.8%) followed by shunt replacement (23.9%). Treatment charges for hydrocephalus were $332 million in 2009 and $418 million in 2013 nationally. High-volume hospitals had more routine discharges compared with lower-volume hospitals (65.7% vs. 50.9%, p<0.0001), which was a trend that improved over time. Multivariate analysis confirmed that hospital volume was independently associated with routine disposition after adjusting for other factors such as patient age, length of stay, and shunt type. However, hospital volume showed a small association with length of stay (β = -0.05, p = 0.0001) and did not impact hospital charges on multivariate analysis.

Conclusion

This analysis provides a recent update of hydrocephalus epidemiology, trends, and outcomes nationally. Estimates from this study suggest that hydrocephalus is a common and costly problem. Hospital volume was for the first time to be associated with important differences in patient outcomes.

## Introduction

Hydrocephalus is a heterogeneous, multifactorial disease resulting from a dynamic imbalance of cerebrospinal fluid (CSF) production and absorption [1]. Regardless of disease etiology, the treatment of hydrocephalus by shunting remains a common strategy. Prior studies have suggested that CSF diversion procedures account for $2-8 billion annually of hospital costs in the U.S. [2, 3] Even with modern biomedical advancements in shunts that increase favorable outcomes, half of all ventricular shunts require revision in the first year, resulting in more surgical procedures with a higher risk of complications [4-7]. Despite how common hydrocephalus is among neurosurgery patients, there is insufficient analysis in the literature of the epidemiology of shunted hydrocephalus as well as the impact of hospital volume on outcomes. The purpose of this study was to use the National Inpatient Sample (NIS) database to better understand the incidence of disease, treatment trends, costs, and impact of hospital volume on the outcomes of CSF shunts in a large population in the U.S.

## Materials and methods

The NIS database, developed by the Healthcare Cost and Utilization Project and funded by the Agency for Healthcare Research and Quality [8, 9], is a nationwide, stratified sample of approximately 20% of inpatient hospital stays including adult and pediatric patients. We used the database to evaluate patients admitted to U.S. hospitals in 2009, 2011, and 2013. Patients treated shunted hydrocephalus were identified with a query using International Classification of Diseases, Ninth Revision, Clinical Modification codes 62190 (creation of shunt; subarachnoid/subdural-atrial, -jugular, -auricular), 62192 (creation of shunt; subarachnoid/subdural-peritoneal, -pleural, other terminus), 62220 (creation of shunt; ventriculo-peritoneal, -pleural, other terminus), 62256 (removal of complete cerebrospinal fluid shunt system; without replacement), 62258 (removal of complete cerebrospinal fluid shunt system; with replacement by similar or other shunt at same operation), 62194 (replacement or irrigation, subarachnoid/subdural catheter), 62225 (replacement or irrigation, ventricular catheter), 62230 (replacement or revision of cerebrospinal fluid shunt, obstructed valve, or distal catheter in shunt system), 0397 (revision of spinal thecal shunt), 0398 (removal of spinal thecal shunt), and 3405 (creation of pleuroperitoneal shunt). Different types of shunts were factored separately in the analysis. Patients were weighted using a database-encoded trend weight variable (i.e., DISCWT), which adjusts for a stratified clustered sampling of each year’s data, allowing for regional and national estimates [2].

The primary outcomes were length of stay, routine disposition, and charges. Patients were divided into those that receive ventriculoperitoneal (VP), ventriculoatrial, ventriculopleural, lumboperitoneal, or other shunts as well as those who had shunt replacement or removal. Hospital volumes were distinguished into low, middle, and high tiers based on evenly distributing case volumes into thirds for each year. Routine disposition was categorized for patients with home or home health discharges. Hospital charges for all years were adjusted to 2009 levels via the Bureau of Labor Statistics medical price inflation index. Hospital charges were calculated for all patients as well as patients where a shunt procedure was the primary reason for hospitalization.

Mean ± standard deviations are provided for continuous measures. T-tests were used to evaluate continuous variables, while Chi-squared tests were used for discrete variables. Linear and logistic regression were used for the evaluation of continuous or discrete outcomes, respectively, with univariable factors that showed a p<0.2 subsequently entered into multivariable models. Multivariate models aimed to adjust for patient demographics (e.g., patient age) as well as surgical variables (e.g., shunt type). Two-way analysis of variance (ANOVA) with main interaction models was performed for the analysis of outcomes across years. With the known limitations of generating comparisons using large databases, a p<0.0001 was considered, but more importantly, effect size was used to interpret the results [10, 11]. Statistical analysis was performed using SPSS V26.0 (IBM Corp., Armonk, NY, USA). The Strengthening the Reporting of Observational Studies in Epidemiology guidelines were used for the drafting and completion of this paper.

## Results

Demographics and outcomes

**Figure 1:**
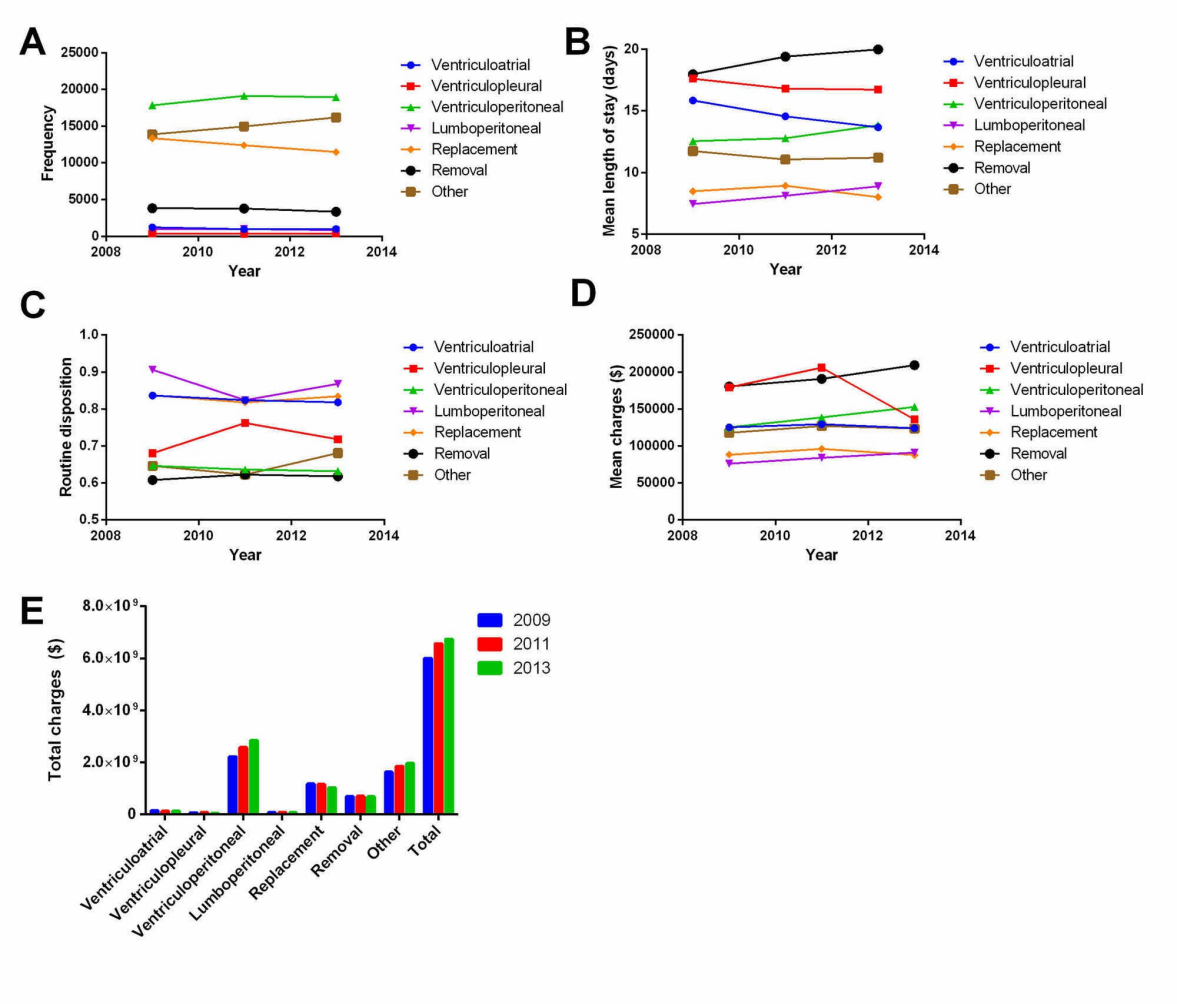
Trends in shunt types, length of stay, disposition, and charges over time assessed with two-way ANOVA A) Numbers of VP shunts increased relative to other types over time (34.7% in 2009 to 36.4% in 2011). Rates of shunt replacements or removals decreased over time. B) Length of stay increased for VP and lumboperitoneal shunt placement, as well as shunt removal relative to other types over time (p<0.0001). C) Routine disposition showed some significant year-to-year fluctuation (p<0.0001), but clinically relevant differences were low. D) Mean charges increased most consistently for VP shunt replacements or removals (p<0.0001). E) Total cumulative charges from 2009 to 2013 are shown for various shunt types. VP, ventriculoperitoneal; ANOVA, analysis of variance

**Figure 2:**
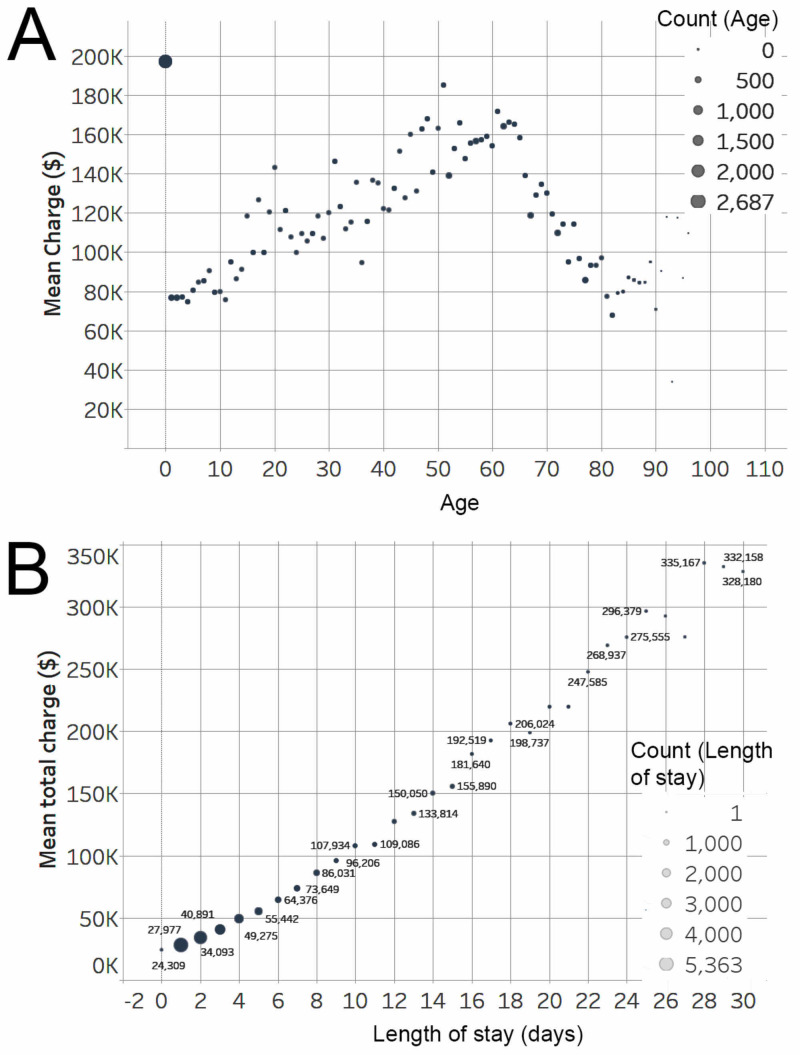
Distribution of cost by age and length and stay A) Comparison of age and mean charge shows significant outliers driven by large numbers of patients <1 year of age. Patients >1 year up to 100 years show mean charges ranges from $80,000 to $180,000. B) A view of lengths of stay <30 days demonstrated costs ranging from $24,309 to $64,378 for patient lengths of stay 1–7 days, most likely patients with more straightforward shunt placements or replacements.

A total of 156,205 patients from 2009, 2011, and 2013 were included, with approximately equal numbers of patients each year (Table 1, Figure 1). Rates of VP shunt usage increased from 34.7% of cases in 2009 to 36.4% in 2011 and 36.3% in 2013 (Figure 1A). A decrease in shunt replacements was seen from 2009 (26%) to 2013 (22%). The mean length of stay remained stable for all procedure types over time but increased for removal, VP shunts, and lumboperitoneal shunts (Figure 1B). Disposition status also remained fairly stable from year to year, with low clinically relevant differences albeit with some year-to-year fluctuations for different shunt types (Figure 1C). The mean/median charges per patient for shunts increased from 2009 ($117,529/$48,925) to 2013 ($132,206/$65,795) (Figure 1D); furthermore, patients undergoing VP shunt placement or shunt removal had a more rapid increase in costs over time compared with other shunt placements (Figure 1D). There was a sharp drop in costs for ventriculopleural shunts in 2013. When factoring only primary shunt procedures, the mean/median charges per patient also increased from 2009 ($62,189/$36,516) to 2013 ($82,172/$45,856). In 2009, 2011, and 2013, treatment of hydrocephalus with its associated diseases accounted for $5.9 billion, $7.0 billion, and $7.8 billion (Figure 1E), respectively. Treatment of hydrocephalus alone accounted for $332 million, $390 million and $420 million, respectively. The distribution of patient age and cost demonstrate significant heterogeneity with outlier costs for patients aged <1 year (i.e., neonatal patients) (Figure 2). Pediatric patients were more likely to undergo shunt replacement (37.4% vs. 18.4%, p=0.0001) than adults and more likely to be treated in small/medium, urban teaching hospitals (p=0.0001)(Table 2). In addition, pediatric patients were more likely for home disposition (84.8 vs 49.3%, p=0.0001).

**Table 1:** Impact of hospital volume on outcomes for hydrocephalus Values indicate number of patients (percentage) unless otherwise indicated. SNF, skilled nursing facility

	Lowest volume n = 52945	Mid volume n = 52085	Highest volume n = 51174	P-value
Age, years (mean ± SD)	51.6 ± 23.5	37.5 ± 27.4	31.6 ± 26.6	<0.0001
Pediatric patients (<18 years)	5052 (9.6)	17029 (32.8)	21776 (42.7)	<0.0001
Admission source				<0.0001
Routine/birth/other	4521 (58.0)	4009 (48.8)	3092 (58.5)	
Emergency room	2397 (30.8)	3230 (39.3)	1257 (23.8)	
Other hospital	581 (7.5)	567 (6.9)	904 (17.1)	
Other facility	274 (3.5)	409 (5.0)	35 (0.7)	
Court/law	19 (0.2)	5 (0.1)	0 (0)	
Admission type				<0.0001
Emergency	10939 (37.1)	12761 (42.8)	11783 (38.9)	
Urgent	4701 (16.0)	6175 (20.7)	8819 (29.1)	
Elective	13082 (44.4)	10079 (33.8)	8756 (28.9)	
Newborn	367 (1.2)	442 (1.5)	492 (1.6)	
Trauma	370 (1.3)	328 (1.1)	433 (1.4)	
Length of stay, days (mean ± SD)	11.5 ± 18.5	12.6 ± 20.8	11.5 ± 19.0	<0.0001
Primary payer				<0.0001
Medicare	21974 (41.6)	13732 (26.4)	10467 (20.5)	
Medicaid	8956 (33.8)	14299 (27.5)	16044 (31.4)	
Private	17830 (33.8)	19290 (37.1)	20953 (41.0)	
Self-pay	2091 (4.0)	2182 (4.2)	1460 (2.9)	
No charge	233 (0.4)	242 (0.5)	73 (0.1)	
Other	1737 (3.3)	2300 (4.4)	2078 (4.1)	
Shunt type				<0.0001
Ventriculoatrial	758 (1.4)	1089 (2.1)	1341 (2.6)	
Ventriculopleural	278 (0.5)	437 (0.8)	351 (0.7)	
Ventriculoperitoneal	21641 (40.9)	18412 (35.3)	15859 (31.0)	
Lumboperitoneal	1163 (2.2)	853 (1.6)	766 (1.5)	
Replacement	9968 (18.8)	13326 (25.6)	13966 (27.3)	
Removal	3322 (6.3)	3702 (7.1)	3909 (7.6)	
Other	15814 (29.9)	14264 (27.4)	14983 (29.3)	
Charges (mean ± SD)	122311 ± 202061	127175 ± 201485	128400 ± 201246	<0.0001
Hospital bed size				<0.0001
Small	5114 (9.8)	5412 (10.9)	770 (1.5)	
Medium	15718 (30.0)	6092 (12.3)	5386 (10.5)	
Large	31604 (60.3)	38159 (76.8)	45018 (88.0)	
Hospital owners				<0.0001
Gov or private-nonprofit	46661 (89.0)	48166 (97.0)	50599 (98.9)	
Private invest-owned	5776 (11.0)	1498 (3.0)	575 (1.1)	
Hospital teaching status				<0.0001
Rural	1885 (3.6)	849 (1.7)	0 (0)	
Urban non-teaching	19108 (36.4)	3357 (6.8)	0 (0)	
Urban teaching	31444 (60.0)	45458 (91.5)	51174 (100)	
Hospital region				<0.0001
Northeast	8419 (15.9)	8777 (16.9)	8937 (17.5)	
Midwest	11268 (21.3)	13152 (25.3)	12331 (24.1)	
South	19863 (37.5)	19321 (37.1)	15893 (31.1)	
West	13395 (25.3)	10836 (20.8)	14014 (27.4)	
Disposition				<0.0001
*Routine*				
Home	26899 (50.9)	32157 (61.8)	33614 (65.7)	
Home health care	5828 (11.0)	5259 (10.1)	4670 (9.1)	
Non-routine				
SNF, rehab, or other facility	17636 (33.4)	12582 (24.2)	10926 (21.3)	
Against medical advice	83 (0.2)	36 (0.1)	19 (0.04)	
Died in hospital	2347 (4.4)	1999 (3.8)	1919 (3.7)	
Discharged alive, destination unknown	45 (0.1)	10 (0.02	25 (0.05)	
Year				<0.0001
2009	17094 (32.3)	17730 (34.0)	16647 (32.5)	
2011	17415 (32.9)	18286 (35.1)	16878 (33.0)	
2013	18435 (34.8)	16070 (30.9)	17650 (34.5)	

**Table 2:** Subgroup analysis of pediatric shunted patients

	Pediatric patients (<18 years) N=43858	Adult patients (>19 years) N=111843	P-value
Length of stay, days (mean ± SD)	13±26	11±16	0.0001
Shunt type			0.0001
Ventriculoatrial	1554 (3.5)	1629 (1.5)	
Ventriculopleural	261 (0.6)	793 (0.7)	
Ventriculoperitoneal	11926 (27.2)	43895 (39.2)	
Lumboperitoneal	364 (0.8)	2413 (2.2)	
Replacement	16411 (37.4)	20618 (18.4)	
Removal	2823 (6.4)	8096 (7.2)	
Other	10519 (24.0)	34398 (30.8)	
Charges (mean ± SD)	120371±232338	128394±188643	0.0001
Hospital bed size			0.0001
Small	5066 (11.8)	6141 (5.6)	
Medium	9485 (22.1)	17525 (16.0)	
Large	28409 (66.1)	86145 (78.4)	
Hospital teaching status			0.0001
Rural	360 (0.1)	2366 (2.2)	
Urban non-teaching	1650 (3.8)	20712 (18.9)	
Urban teaching	40950 (95.3)	86733 (79.0)	
Hospital region			0.0001
Northeast	6016 (13.7)	20103 (18.0)	
Midwest	10354 (23.6)	26323 (23.5)	
South	16293 (37.2)	38566 (34.5)	
West	11194 (25.5)	26850 (24.0)	
Disposition			0.0001
Routine			
Home	37198 (84.8)	55081 (49.3)	
Home health care	3099 (7.1)	12636 (11.3)	
Non-routine			
SNF, rehab, or other facility	2821 (6.4)	38308 (34.3)	
Against medical advice	10 (0.02)	128 (0.1)	
Died in hospital	720 (1.6)	5536 (5.0)	
Discharged alive, destination unknown	10 (0.02)	70 (0.1)	

The top diagnoses associated with specific shunt procedures were expected and consistent from 2009 to 2013 (Table 3). These included mechanical complications of shunts (966.2), idiopathic normal pressure hydrocephalus (331.5), obstructive hydrocephalus (331.5), infection (996.63), and subarachnoid hemorrhage (430). Disease of hard tissues of teeth (521) was also associated with hydrocephalus but suggested a high rate of incidental findings on head imaging.

**Table 3:** Top diagnoses and International Classification of Disease 9 codes associated with hydrocephalus treatment

Ventriculoatrial	Ventriculopleural	Ventriculoperitoneal	Replacement	Removal
Mechanical complication of nervous system device, implant, and graft (966.2)	Mechanical complication of nervous system device, implant, and graft (966.2)	Idiopathic normal pressure hydrocephalus (331.5)	Mechanical complication of nervous system device, implant, and graft (966.2)	Infection and inflammatory reaction due to nervous system device, implant, and graft (996.63)
Diseases of hard tissues of teeth (521)	Idiopathic normal pressure hydrocephalus (331.5)	Obstructive hydrocephalus (331.4)	Other complications due to nervous system device, implant, and graft (996.75)	Mechanical complication of nervous system device, implant, and graft (966.2)
Infection and inflammatory reaction due to nervous system device, implant, and graft (996.63)	Infection and inflammatory reaction due to nervous system device, implant, and graft (996.63)	Communicating hydrocephalus (331.3)	Infection and inflammatory reaction due to nervous system device, implant, and graft (996.63)	Subarachnoid hemorrhage (430)

Hospital volume and length of stay

**Figure 3:**
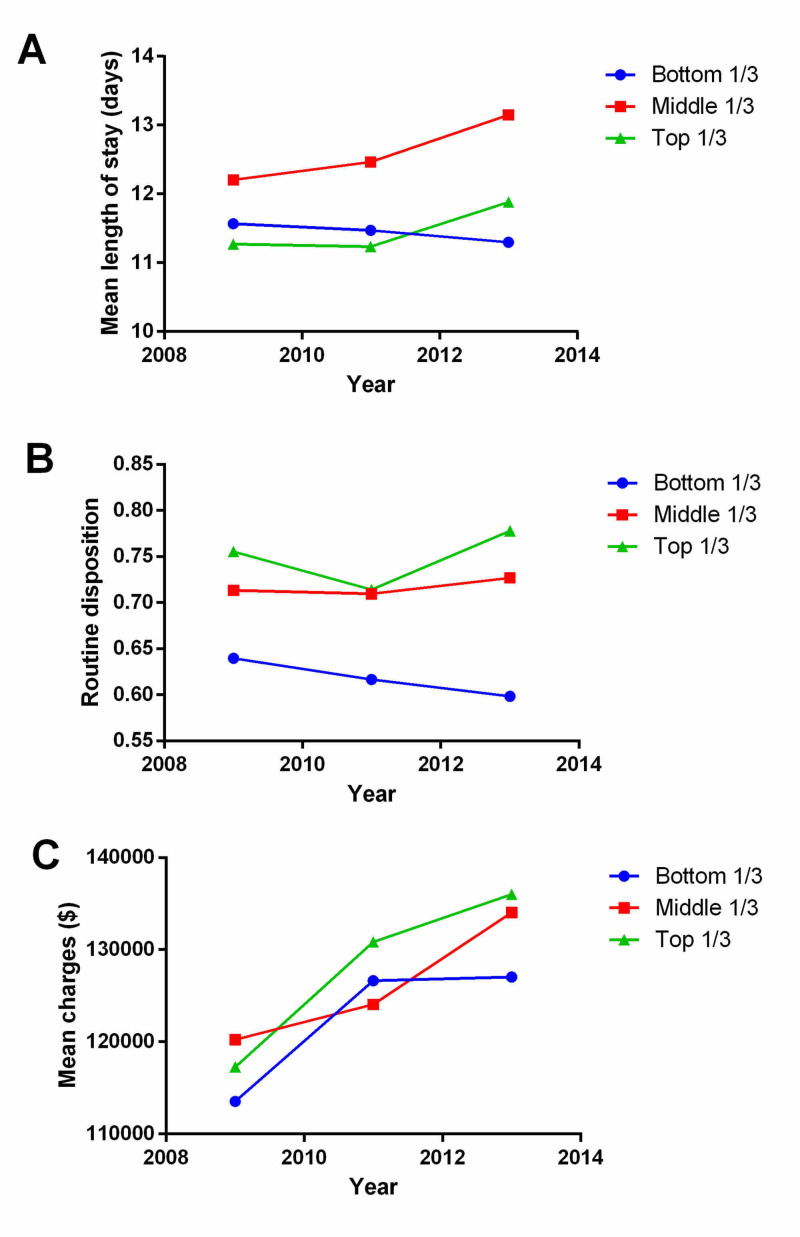
Evaluation of outcomes by hospital volumes assessed by two-way ANOVA A) Length of stay was similar in low- and high-volume centers but was longer at mid-volume hospitals (p<0.0001). B) Mid- and high-volume centers showed a greater association with routine disposition (p<0.0001). C) Mean costs were statistically significantly but not clinically different depending on hospital volume (p<0.0001). Mean costs showed an increase regardless of hospital volume and remained stable from 2011 to 2013 for lower-volume centers. ANOVA, analysis of variance

We compared variables across low-, mid-, and high-volume hospitals (Table 1, Figure 3). A significantly younger population was treated in high-volume compared with medium- or low-volume hospitals (31.6 ± 26.6 years vs. 37.5 ± 27.4 years vs. 51.6 ± 23.5 years, p<0.0001), likely reflecting the care of neonatal patients in high-volume centers. High-volume hospitals also had a substantial portion of patients admitted from other hospitals (17.1%) and under emergency (38.9%) or urgent (29.1%) circumstances compared with low- and mid-volume hospitals. Length of stay was not substantially different between low- and high-volume hospitals but was higher for mid-volume hospitals (Figure 3A). High-volume hospitals had more beds, were primarily government or private-nonprofit funded, and were uniformly urban teaching hospitals. High-volume centers were predominantly in the South and West. Routine disposition (e.g., home or home health) was more likely in high-volume hospitals (74.8%) compared with low- (61.9%) or mid-volume (71.9%) hospitals (p<0.0001) (Figure 3B). Between 2009 and 2013, overall mean charges increased, although, for low-volume hospitals, the mean charges appear to remain fairly constant between 2011 and 2013 (Figure 2C).

Multivariable analysis

Multivariate linear and logistic regression of hydrocephalus outcomes was performed to identify factors associated with length of stay, routine discharge, and charges (Tables 4-6). Multivariate regression showed that admission source (β = -0.26, p = 0.0001) and shunt type (β = -0.11, p = 0.0001) were associated with length of stay (Table 4). Hospital volume did not impact the length of stay. Multivariate logistic regression suggested that admission via the emergency room (odds ratio [OR] = 0.32, p = 0.0001) or other facility (OR = 0.23, p = 0.0001) was associated with lower rates of routine disposition (Table 5). Similarly, each of the payer types, except self-payers, showed lower rates of routine disposition (range OR = 0.73-0.84) compared with no charge patients (OR = 3.4). Although all shunt types were associated with routine disposition, ventriculopleural (OR = 2.88) and lumboperitoneal (OR = 1.98) shunts showed the highest association with routine disposition. Interestingly, the placement of a VP shunt (OR = 1.16) showed a lower association with routine disposition compared with shunt replacement (OR = 1.5). Middle- and high-volume hospitals showed a greater association with routine disposition while low-volume hospitals failed to show a strong association with routine disposition after adjusting for other factors. Multivariate linear regression showed that only length of stay was significantly associated with hospital charges (β = 0.75, p = 0.0001) (Table 6).

**Table 4:** Evaluation of factors affecting length of stay

	Univariate		Multivariate	
	Standardized β value	P-value	Standardized β value	P-value
Age	-0.067	0.0001	-0.065	0.0001
Admission source	-0.2	0.0001	-0.26	0.0001
Admission type	-0.074	0.0001	0.007	0.6
Primary payer	0.037	0.0001	-0.025	0.04
Shunt type	-0.027	0.0001	-0.11	0.0001
Hospital bed size	0.015	0.0001	-0.048	0.0001
Hospital owners	-0.009	0.0001	-0.004	0.7
Hospital teaching status	0.054	0.0001	0.063	0.0001
Hospital region	-0.016	0.0001	0.022	0.09
Hospital volume	-0.001	0.8		
Year	0.008	0.001		

**Table 5:** Evaluation of factors affecting routine disposition OD, odds ratio; CI, confidence interval

	Univariate		Multivariate	
	OR (95% CI)	P-value	OR (95% CI)	P-value
Age	0.965 (0.964, 0.965)	0.0001	0.962 (0.960, 0.964)	0.0001
Admission source				
Emergency room	0.36 (0.33, 0.38)	0.0001	0.32 (0.3, 0.35)	0.0001
Other hospital	0.22 (0.2, 0.24)	0.0001	0.23 (0.21, 0.26)	0.0001
Other facility	0.13 (0.11, 0.15)	0.0001	0.13 (0.1, 0.15)	0.0001
Court/law	0.34 (0.15, 0.75)	0.008	0.43 (0.17, 1.11)	0.08
Routine/birth/other	Reference		Reference	
Admission type				
Emergency	1.76 (0.49, 6.4)	0.4		
Urgent	1.86 (0.51, 6.73)	0.3		
Elective	3.62 (0.998, 13.13)	0.05		
Newborn	7.22 (1.97, 26.46)	0.003		
Trauma	0.28 (0.08, 1.03)	0.06		
Other	Reference			
Length of stay	0.97 (0.967, 0.968)	0.0001	0.96 (0.958, 0.962)	0.0001
Primary payer				
Medicare	0.38 (0.36, 0.4)	0.0001	0.73 (0.6, 0.91)	0.004
Medicaid	1.43 (1.34, 1.52)	0.0001	0.75 (0.61, 0.92)	0.006
Private	1.07 (1.006, 1.14)	0.03	0.77 (0.63, 0.92)	0.01
Self-pay	0.86 (0.79, 0.93)	0.0001	0.84 (0.63, 1.11)	0.2
No charge	0.997 (0.817, 1.217)	0.98	3.4 (1.47, 7.98)	0.004
Other	Reference		Reference	
Shunt type				
Ventriculoatrial	2.94 (2.18, 2.62)	0.0001	1.28 (0.995, 1.652)	0.06
Ventriculopleural	1.37 (1.2, 1.67)	0.0001	2.88 (1.76, 4.69)	0.0001
Ventriculoperitoneal	0.94 (0.91, 0.96)	0.0001	1.16 (1.07, 1.27)	0.001
Lumboperitoneal	3.39 (3.03, 3.78)	0.0001	1.98 (1.44, 2.71)	0.0001
Replacement	2.63 (2.55, 2.72)	0.0001	1.5 (1.35, 1.67)	0.0001
Removal	0.82 (0.79, 0.86)	0.0001	0.89 (0.78, 1.02)	0.09
Other	Reference		Reference	
Charges	-	-		
Hospital size				
Small	1.77 (1.69, 1.86)	0.0001	1.64 (1.41, 1.91)	0.0001
Medium	1.23 (1.19, 1.26)	0.0001	1.09 (0.98, 1.21)	0.1
Large	Reference		Reference	
Hospital owners				
Gov or private	1.38 (1.32, 1.45)	0.0001	1.49 (1.1, 1.91)	0.0001
Private	Reference		Reference	
Hospital teaching status				
Rural	0.82 (0.76, 0.89)	0.0001	1.14 (0.89, 1.44)	0.3
Urban non-teaching	0.66 (0.64, 0.67)	0.0001	1.08 (0.98, 1.18)	0.1
Urban teaching	Reference			
Hospital region				
Northeast	0.66 (0.64, 0.68)	0.0001	0.57 (0.5, 0.64)	0.0001
Midwest	0.85 (0.83, 0.88)	0.0001	0.52 (0.43, 0.62)	0.0001
South	1.09 (1.06, 1.12)	0.0001	0.77 (0.69, 0.86)	0.0001
West	Reference			
Hospital volume				
Low	0.54 (0.53, 0.56)	0.0001	0.99 (0.88, 1.12)	0.9
Medium	0.86 (0.84, 0.88)	0.0001	1.27 (1.13, 1.43)	0.0001
High	Reference		Reference	
Year				
2009	1.02 (0.99, 1.05)	0.1		
2011	0.92 (0.87, 0.94)	0.0001		
2013	Reference			

**Table 6:** Evaluation of factors affecting charges

	Univariate		Multivariate	
	Standardized β value	P-value	Standardized β value	P-value
Age	-0.01	0.0001	-0.005	0.5
Admission source	-0.26	0.0001	-0.1	0.0001
Admission type	-0.069	0.0001	0.039	0.0001
Length of stay	0.8	0.0001	0.75	0.0001
Primary payer	0.048	0.0001	0.053	0.0001
Shunt type	-0.019	0.0001	0.036	0.0001
Hospital size	0.023	0.0001	0.052	0.0001
Hospital owners	0.039	0.0001	0.003	0.6
Hospital teaching status	0.045	0.0001	0.063	0.0001
Hospital region	0.025	0.0001	-0.132	0.0001
Hospital volume	0.012	0.0001	-0.05	0.0001
Year	0.03	0.0001	0.009	0.3

## Discussion

National trends

**Figure 4:**
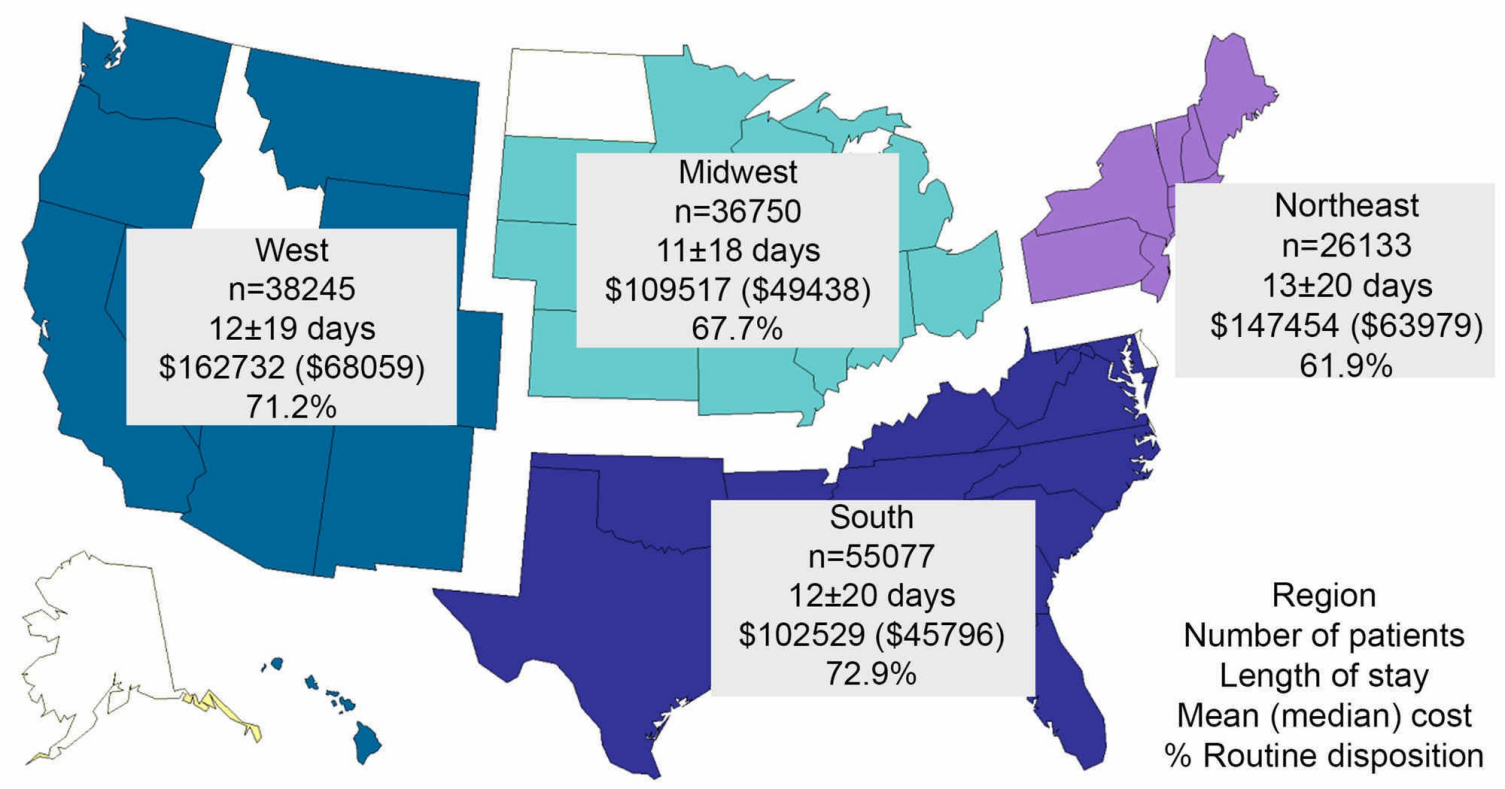
Hydrocephalus outcomes by hospital regions The total numbers of surgically treated hydrocephalus cases from 2009 to 2013 are shown along with mean length of hospital stay, mean charges, and rates of routine disposition. Lower mean charges and higher rates of routine disposition were more likely in the South compared with other regions, which correlated with the higher prevalence of high-volume centers in this region.

These results help inform regarding the number of procedures used to treat hydrocephalus annually (~50,000) and the net cost of this disease ($418 million in 2013). These results also help break down patient characteristics, hospital types, and regional differences related to hydrocephalus treatment. High-volume hospitals did not have a substantially lower length of stay or lower charges, but despite having a higher rate of urgent and emergent admission sources, they did show higher rates of routine disposition. In addition, the greater likelihood of routine disposition in high-volume hospitals increased over time compared with the lowest volume hospitals. High-volume hospitals were also more prevalent in the South and West, suggesting geographic variation in patient outcomes and access to care. Evaluation of hospital regions showed that hospitals in the South were more likely to have lower mean charges and greater routine disposition compared with other regions (Figure 4). These findings remained evident in multivariate analysis which attempted to account for some patient heterogeneity.

Epidemiology of hydrocephalus treatment

Various studies have aimed to evaluate the incidence, prevalence, and cost of hydrocephalus treatment. Most have used single-center studies evaluating specific etiologies of hydrocephalus [7, 12]. Bondurant et al. [13] reported one of the first database studies in 1995, citing a prevalence of 125,000 shunts in the U.S., 69,000 discharges annually with hydrocephalus diagnosis, 36,000 annual shunt-related procedures, and an annual expenditure of $100 million per year. These authors admitted the challenges of identifying accurate epidemiological data for hydrocephalus treatment, and their results are likely underestimates of the true incidence and cost. Another study estimating rates of normal pressure hydrocephalus suggested an incidence of 1.2:1000 inhabitants annually, which was much higher than prior estimates [14]. Interestingly, this study predicted that the incidence of shunt-related surgery for normal pressure hydrocephalus was between 1 and 2:100,000 inhabitants annually, suggesting a highly underdiagnosed and undertreated disease. The underdiagnosis of normal pressure hydrocephalus has been shown in other large studies [15]. More recent studies have aimed to use multicenter registries to evaluate hydrocephalus incidence and complication profiles [16, 17].

Global estimates of hydrocephalus suggest a higher incidence - 145:100,000 births in Africa and 316:100,000 births in Latin America compared with 68:100,000 births in the U.S. using data from a meta-analysis [18]. That study predicted 400,000 new cases of pediatric hydrocephalus annually with disproportionate rates in low- and middle-income countries. Others have verified the challenges for global surgery in addressing pediatric hydrocephalus [19, 20].

The most recent study from the NIS in 2000 identified 5,574 admissions and helped characterize shunts types, hospital characteristics, and costs [2]. Most shunts were VP shunts (43.4%), while most admissions were elective (43.3%) for treatment of shunt malfunction (40.7%). A mean cost of $35,816 was identified per case, which predicted a $1.1 billion annual cost in the U.S. for treatment. Approximately 20% of cases cost more than $50,000, and 10% cost more than $100,000, suggesting inclusion of other diagnostic codes similar to our series. Nonetheless, hydrocephalus treatment accounted for significant healthcare expenditure. Our results further clarify this study by also studying primary shunt procedures alone, data over recent years, and involved a more comprehensive list of International Classification of Diseases 9 codes for including and studying patients. In addition, our results are the first to consider the impact of hospital volume on patient impact.

Outcomes and healthcare disparities by hospital characteristics

Variation in hospital volume, type, and region had impacts on hydrocephalus treatment despite shunts being a common procedure across all neurosurgery. In a series of 373 pediatric patients treated for hydrocephalus, Walker et al. [21] showed that those with public insurance stayed in the hospital an average of five days longer and were more likely to be transferred from other hospitals, to be born prematurely, and to present with a neoplasm. These results suggest some socioeconomic disparity in access to hydrocephalus treatment. Our results help to explore some of the regional differences in treatment and suggest that admissions via the emergency room and birth admission, in particular, were associated with lower length of stay, which may suggest that earlier treatment may improve outcomes. However, the younger mean age of patients in high-volume hospitals suggests that the care for neonatal patients occurs in these institutions. Despite the increased complexity of these patients, higher-volume hospitals were still overall able to achieve higher rates of routine disposition. Accessibility of hydrocephalus and other disease treatment in urban teaching institutions and by geographic region has been shown in other studies [2, 4, 22].

Limitations

Limitations of this study mainly surround the use of an administrative database. Although this information provides population-level estimates, variables are dependent on coding and may include heterogeneous groups of patients. Our estimates of mean, median, and total cost for hydrocephalus treatment vary from some prior studies. Several reasons for this may be that this study has been more inclusive of different treatment modalities, includes a database predicting the national population, and involves the use of hospital charges. Further economic modeling would be needed to more accurately determine the cost of hydrocephalus treatment. One of the challenges with this data involved the heterogeneity of diagnoses requiring shunt treatment due to the use of multiple diagnostic codes within the NIS for each individual patient. Additionally, P-values in our study were frequently significant at a 0.05 level, likely because of the very large sample sizes in this study. With the known limitations with generating comparisons using large databases, a p<0.0001 was considered, but more importantly, effect size was used to interpret the results. Rather than simply using P-values, we utilized the effect size, including β coefficients, in multivariate analysis to analyze group differences.

## Conclusions

This analysis provides a recent update of hydrocephalus epidemiology, trends, and outcomes nationally. Shunt treatment for hydrocephalus involves up to 50,000 cases a year. In addition, hydrocephalus treatment is implicated alone or in combination with other diseases to a cost of $418 million annually in the U.S. Despite being a relatively common disease, hydrocephalus treatment and patient outcomes are impacted by types of hospitals and surgeon volume. Even with more complex cases in higher volume centers, routine disposition was more common. In addition, the increased complexity of neonatal care and hydrocephalus management has likely also pushed towards the increased treatment of hydrocephalus in tertiary care centers. Nonetheless, hydrocephalus treatment remains one of the most common procedures in neurosurgery where the availability of surgical treatment options and management in lower-volume (e.g., community) hospitals will be critical to meeting the need of this patient population. These data suggest that hospital volume does impact care and that there is an opportunity for improving gaps in outcomes. Further study is needed to identify sources of these gaps and potentially improve patient treatment.
